# Metabolomics and transcriptomics analyses for characterizing the alkaloid metabolism of Chinese jujube and sour jujube fruits

**DOI:** 10.3389/fpls.2023.1267758

**Published:** 2023-09-18

**Authors:** Xiaofang Xue, Ailing Zhao, Yongkang Wang, Haiyan Ren, Wanlong Su, Yi Li, Meijuan Shi, Li Liu, Dengke Li

**Affiliations:** Pomology Institute, Shanxi Agricultural University, Shanxi Key Laboratory of Germplasm Improvement and Utilization in Pomology, Taiyuan, China

**Keywords:** Chinese jujube, sour jujube, fruits, metabolome, transcriptome, alkaloid metabolites, candidate genes, regulatory network

## Abstract

**Introduction:**

Jujube is an important economic forest tree whose fruit is rich in alkaloids. Chinese jujube (*Ziziphus jujuba* Mill.) and sour jujube (*Ziziphus spinosa* Hu.) are the two most important species of the jujube genus. However, the mechanisms underlying the synthesis and metabolism of alkaloids in jujube fruits remain poorly understood.

**Methods:**

In this study, the fruits of *Ziziphus jujuba* ‘Hupingzao’ and *Ziziphus spinosa* ‘Taigusuanzao’ in different harvest stages were used as test materials, we first integrated widely targeted metabolomics and transcriptomics analyses to elucidate the metabolism of alkaloids of jujube fruits.

**Results:**

In the metabolomics analysis, 44 alkaloid metabolites were identified in 4 samples, 3 of which were unique to sour jujube fruit. The differential alkaloid metabolites (DAMs) were more accumulated in sour jujube than in Chinese jujube; further, they were more accumulated in the white ripening stage than in the red stage. DAMs were annotated to 12 metabolic pathways. Additionally, transcriptomics data revealed 259 differentially expressed genes (DEGs) involved in alkaloid synthesis and metabolism. By mapping the regulatory networks of DAMs and DEGs, we screened out important metabolites and 11 candidate genes.

**Discussion:**

This study preliminarily elucidated the molecular mechanism of jujube alkaloid synthesis. The candidate genes regulated the synthesis of key alkaloid metabolites, but the specific regulation mechanism is unclear. Taken together, our results provide insights into the metabolic networks of alkaloid synthesis in Chinese jujube and sour jujube fruits at different harvest stages, thereby providing a theoretical reference for further research on the regulatory mechanism of jujube alkaloids and their development and utilization.

## 1 Introduction

Chinese jujube (*Ziziphus jujuba* Mill.) and sour jujube (*Ziziphus spinosa* Hu.) are the two most important species of the jujube genus (Qu and Wang, 1991). The cultivated jujube originates from sour jujube (Liu et al., 2010). Chinese jujube is an important economic forest tree originating from China, with rich germplasm resources and a history of more than 7000 years (Liu and Wang, 2009). Chinese jujube fruit is a traditional “medicine and food homology” nourishing product (Guo et al., 2012), which is often considered the initiator of Chinese medicine, as it is rich in various nutritional active ingredients such as sugars (Fu et al., 2021), organic acids (Wang L. N. et al., 2018), flavonoids (Xue et al., 2022), triterpenoids (Wen et al., 2022; Pan et al., 2023), cyclic nucleotides (Jiang et al., 2019), vitamins (Wojdyło et al., 2016), and alkaloids (Rashwan et al., 2020). Studies have shown that Chinese jujube fruit possesses antioxidant (Ren et al., 2022), anti-inflammatory (Goyal et al., 2011), liver protection (Liu et al., 2015), blood pressure and lipid reduction (Dou et al., 2023), and anticancer (Shi et al., 2022) properties. In addition to Chinese jujube, sour jujube is an important species of the jujube genus. Traditionally, the seed kernel is the most valuable part of sour jujube because of its active ingredients such as spinosin and saponin, which promote sleep (Shen et al., 2020; Zhang S. et al., 2020). Moreover, recent studies have shown that sour jujube pulp contains various nutrients, such as flavonoids and vitamin C, in higher concentration than that in Chinese jujube fruits (Sun et al., 2016; Xue et al., 2022). Therefore, the expanded use of sour jujube pulp will help improve the additional value of the jujube industry.

Alkaloids are a type of secondary metabolites widely found in plants (Hegnauer, 1950). They are nitrogenous organic compounds with various structural types and play an important protective role against biological and abiotic stresses (Yahyazadeh et al., 2018). Alkaloids have obvious physiological and pharmacological activities, such as antibacterial, regulating blood lipids, anti-cell proliferation, anti-allergy, anti-inflammatory, which makes them relevant for the research and development of new drugs (Cao et al., 2016; Shang et al., 2018; Bai et al., 2021). Natural plants are important sources of alkaloids. For example, more than 40 alkaloids have been found in oats (*Avena sativa* L.) (Wang D. et al., 2021). Alkaloids are the main active components of mulberry leaves (Wu, 2021). As many as 124 alkaloids have been reported in *Lycium* genus, which are classified into organic, heterocyclic derivative, and other alkaloids (Liu et al., 2022). Moreover, 12 alkaloids were isolated from *Robinia acacia*, which showed antibacterial activity against *Staphylococcus aureus* and *Escherichia coli* (Li and Zhang, 2021).

Similarly, alkaloids are important bioactive substances in jujube. Previous research on jujube alkaloids mainly focused on identification, extraction, and characterization of bioactive activity. First, the identification found that the alkaloids in jujube are mainly distributed in the root bark, trunk bark, seeds, fruits, and leaves. At present, the alkaloids found in jujube mainly include cyclic peptide alkaloids and isoquinoline alkaloids (Wang Y. et al., 2021). The main methods for the determination of alkaloids in jujube are high performance liquid chromatography and liquid chromatography–mass spectrometry (Jiang et al., 2009; Li et al., 2017). Second, the alkaloids in jujube were separated by extraction. The extraction methods of alkaloids mainly include water extraction, alcohol extraction, cold immersion extraction, ultrasonic extraction and supercritical extraction (Zhang et al., 2010), among which organic solvent extraction method is the most widely used method. Finally, the bioactive activity of alkaloids in sour jujube kernel is anti-depressant and sedative (Fu et al., 2005; Zhu et al., 2009), while those from Chinese jujube have antioxidant effects and can clear DPPH free radicals (Zhang et al., 2019).

Despite extensive knowledge on alkaloids in jujube, no research has focused on the synthesis mechanism of jujube alkaloids. Metabolites are the basis of phenotype and can help understand biological processes and their mechanisms more directly and effectively. Based on qualitative and quantitative analyses of metabolites, metabolomics can be used to analyze metabolic pathways and networks (Ren et al., 2020), whereas transcriptomics can comprehensively and rapidly obtain almost all transcripts from a particular organ or tissue of a species in a certain state, reflecting their expression levels (Surget-Groba and Montoya-Burgos, 2010). With the rapid development of omics technology, metabolomics combined with transcriptomics can not only detect differential metabolites but also explain the underlying causes of metabolite changes at the gene level. This combination has been applied to many species, such as apple (Sun et al., 2021), pear (Liu et al., 2021), cherry (Wang Y. et al., 2023), papaya (Zheng et al., 2021), and pitaya (Wu et al., 2022). Zhang et al. studied the color formation mechanism of jujube peel by combining metabolomics and transcriptomics (Zhang Q. et al., 2020). Based on the these findings, the current study aimed to determine the mechanism of alkaloid formation in the fruits of Chinese jujube and sour jujube at different harvest stages using metabolomics combined with transcriptomics. Thus, we can determine the regulatory mechanism of jujube alkaloids and improve their development and utilization in jujube germplasm resources.

## 2 Materials and methods

### 2.1 Plant materials

**Figure 1:**
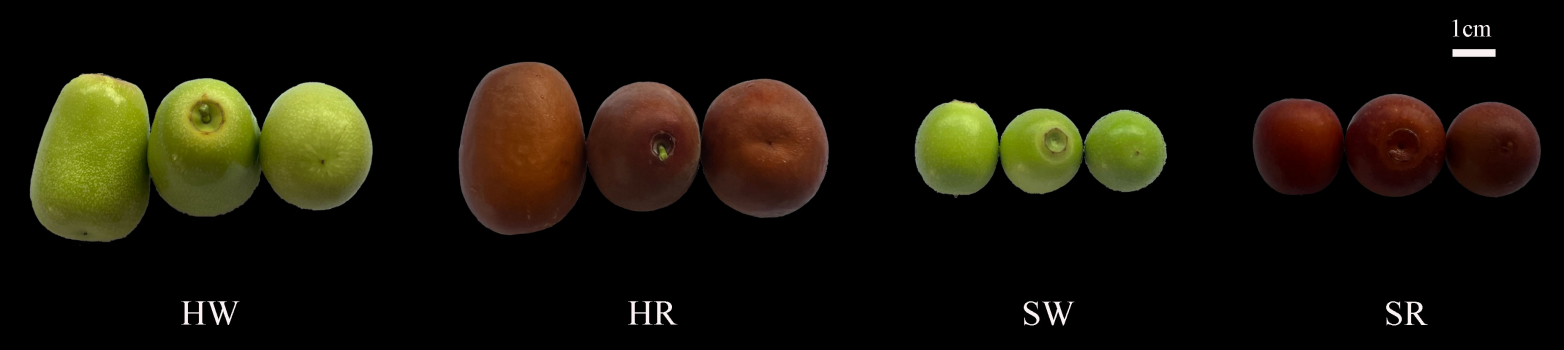
Chinese jujube (*Ziziphus jujuba* ‘Hupingzao’) and sour jujube (*Ziziphus spinosa* ‘Taigusuanzao’) fruits at different harvest stages. HW, HPZ white ripening stage; HR, HPZ red stage; SW, TGSZ white ripening stage; SR, TGSZ red stage.

The experimental materials were collected from the National Jujube Germplasm Repository, Research Institute of Pomology, Shanxi Agricultural University (Taigu, Shanxi, China), the latitude is N37°20’, and the longitude is E112°29’. We selected two important species of the jujube genus, *Ziziphus jujuba* ‘Hupingzao’ (HPZ) and *Ziziphus spinosa* ‘Taigusuanzao’ (TGSZ). The age of the test trees was 30 years; the trees were healthy and showed normal growth under conventional water and fertilizer management conditions. According to the description of the maturity period in the jujube germplasm resources (Li, 2006), fruits were collected from two harvest stages: HPZ white ripening stage (HW) and red stage (HR) as well as TGSZ white ripening stage (SW) and red stage (SR) (**Figure 1**). Each fruit was similar in size, without mechanical damage or disease. Each sample included fruits weighing 500 g. The selected fruits were immediately frozen in liquid nitrogen and stored at −80°C for metabolomics and transcriptomics analyses, with three biological replicates per group, that is, three trees with the same growth of HPZ and TGSZ were selected for sample collection, and each tree was a biological replicate.

### 2.2 Sample preparation and extraction

The collected samples were placed in a lyophilizer (Scientz-100F), vacuum freeze–dried, and crushed using an MM 400 pulverizer (Retsch GmbH, Haan, Germany) for 1.5 min at 30 Hz. Next, 50 mg of powder was weighed and dissolved in 1.2 mL of 70% methanol solution for vortex extraction. This process was repeated 6 times for 30 s, followed by incubation for 30 min. The samples were then centrifuged at 12,000 × *g* for 3 min, after which the supernatant was passed through a 0.22-μm Millipore membrane filter and placed in a liquid sample bottle for ultra-performance liquid chromatography (UPLC) (ExionLC™ AD) and tandem mass spectrometry (MS/MS) analyses.

### 2.3 Analysis of metabolites

The data acquisition instrumentation system consisted of UPLC (ExionLC™ AD) and MS/MS (Applied Biosystems 4500 QTRAP).

The UPLC conditions were as follows: Agilent SB-C18 column (2.1 mm × 100 mm; 1.8 µm); mobile phase, 0.1% (v/v) formic acid water (solvent A) and acetonitrile with 0.1% (v/v) formic acid (solvent B); flow rate, 0.35 mL·min^−1^, injection volume, 5 µL; and column temperature, 40°C. The gradient program was set as follows: 5% B at 0 min, with the proportion of B increasing linearly to 95% within 9 min and remaining constantfor 1 min, after which the proportion decreased to 5% between 10–11 min. The program was equilibrated at 14 min.

The MS conditions were as follows: electrospray ionization source temperature, 550°C; and ion spray voltage, 5500 V (positive mode)/−4500 V (negative mode).Ion source gas I, gas II, and curtain gas were set at 50, 60, and 25.0 psi, respectively, with high values ofthe parameters of collision-induced ionization. QQQ scans were used in multiple reaction monitoring (MRM) mode, and the collision gas (nitrogen) was set to medium. A specific set of MRM ion pairs was monitored during each stage, based on the metabolites eluted during the process.

Based on the Metware database, the material was qualitatively characterized according to the secondary spectrum information. Metabolite quantification was performed using MRM mode analysis of triple four-level rod MS (Wang A. M. et al., 2018). After obtaining the metabolite MS data in different samples, all chromatographic peaks were integrated and the peaks of the same metabolite in different samples were corrected.

To analyze the differences in alkaloids metabolites among the four samples, metabolites with a fold change of ≥2 or ≤0.5 were selected, and metabolites with VIP scores of ≥1 were simultaneously selected (Yuan et al., 2018).

### 2.4 Transcriptome analysis

RNA sequencing and assembly were performed by Beijing BioMarker Biological Technology Co., LTD (Beijing, China). Total RNA was extracted from fruits at HW, HR, SW, and SR stages. RNA concentration and quality were assessed using Agilent 2100 instrument. Then, cDNA libraries were constructed for Illumina sequencing. After removing adapters and low-quality reads, we aligned the clean reads to the reference genome and performed differential expression analysis using the DESeq2 method. The screening conditions for DEGs was log2 foldchange of ≥1 and FDR of <0.05.

### 2.5 Statistical analysis

Data were analyzed using Microsoft Excel v. 2007 (Microsoft Corporation, Redmond, WA, USA). PCA was performed using the Statistical Analysis System v. 9.2 (SAS Institute, Cary, NC, USA). Tbtools (http://doi.org/10.1101/289660) and OriginPro9.0 (OriginLab Corporation, Northampton, MA, USA) were used to construct the figures. The pearson method was used for association analysis of differential alkaloid metabolites and differentially expressed genes, and the values were normalized with log2, with a threshold of 0.8 for association analysis and a *p-value* threshold of 0.05.

## 3 Results

### 3.1 Identification of alkaloid metabolites based on *UPLC-MS* analyses in Chinese jujube and sour jujube fruits

**Figure 2:**
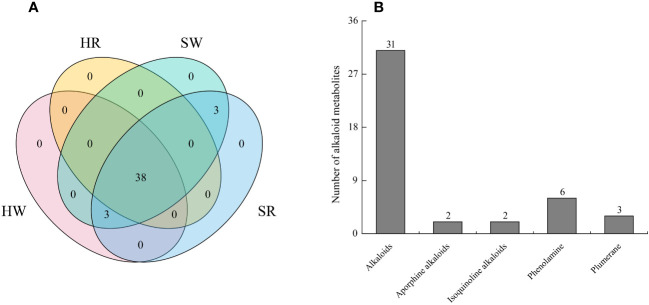
Identification of alkaloid metabolites in Chinese jujube (*Ziziphus jujuba* ‘Hupingzao’) and sour jujube (*Ziziphus spinosa* ‘Taigusuanzao’). **(A)** Venn diagram of alkaloid metabolites in fruits of Chinese jujube and sour jujube at different harvest stages. **(B)** Quantitative analysis of alkaloid metabolites in different classes. HW, HPZ white ripening stage; HR, HPZ red stage; SW, TGSZ white ripening stage; SR, TGSZ red stage.

In this study, we analyzed two important species of the jujube genus, *Ziziphus jujuba* ‘Hupingzao’ (HPZ) and *Ziziphus spinosa* ‘Taigusuanzao’ (TGSZ). A total of 44 alkaloid metabolites were detected in the following 4 samples: HW, HR, SW, and SR samples, as shown in the Venn diagram (**Figure 2A**; **Supplement 1**). Among these 44 metabolites, 38 were detected in all 4 samples. Three metabolites including *N*-p-coumaroylagmatine, frangufoline, and scutianineC were detected only in sour jujube fruits at two different developmental stages. Three metabolites (*N*-acetylputrescine, agmatine, and tryptamine) were not detected in HR samples but were detected in the other three samples. Then, we classified the identified alkaloid metabolites (**Figure 2B**), based on structural differences between metabolites, 44 metabolites could be divided into 5 classes, namely, alkaloids (n = 31), aporphine alkaloids (n = 6), isoquinoline alkaloids (n = 3), phenolamine (n = 2), and plumerane (n = 2).

### 3.2 Analysis of differential alkaloid metabolites (DAMs) of jujube fruits

**Figure 3:**
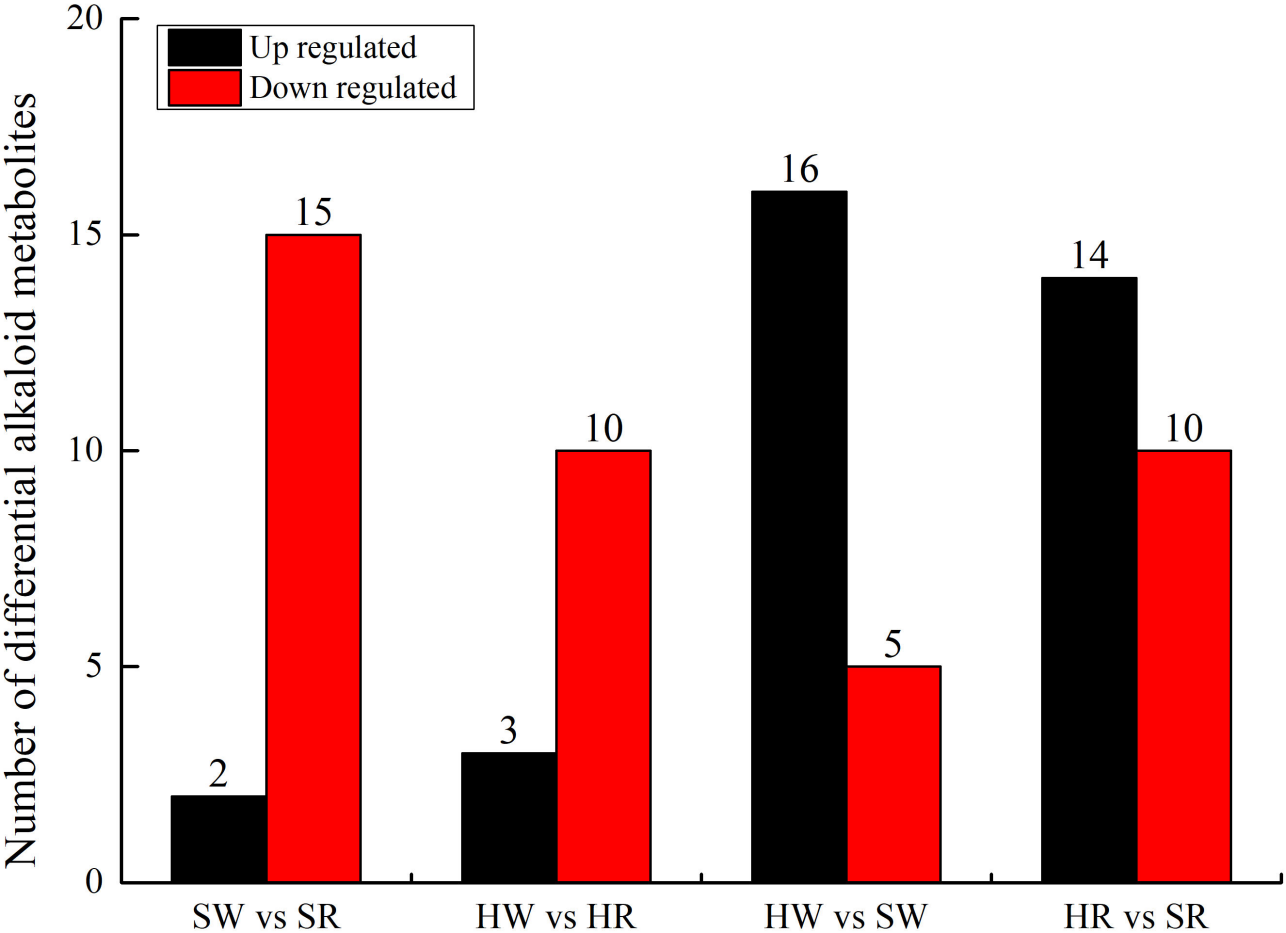
Number of up- and downregulated alkaloid metabolites in various comparison groups. HW, HPZ white ripening stage; HR, HPZ red stage; SW, TGSZ white ripening stage; SR, TGSZ red stage.

A comparative group analysis of SW vs. SR, HW vs. HR, HW vs. SW, and SW vs. SR in four samples (HW, HR, SW, and SR) is shown in **Figure 3**. A total of 34 DAMs were obtained from the 4 comparison groups (**Supplement 2**). In the SW vs. SR comparison group, 17 DAMs were identified; compared with SW, 15 DAMs were down regulated and only 2 DAMs (stepharine and shikonin) were up regulated in SR. In the HW vs. HR comparison group, we identified 13 DAMs; compared with HW, 10 DAMs were down regulated and only 3 DAMs (stepharine, isoboldine, and shikonin) were up regulated in HR. Thus, in both Chinese jujube and sour jujube, DAMs were more accumulated in the white ripening stage than in red stage. These metabolites mainly included *N*-feruloylagmatine, *N*-acetylputrescine, jubanineA, frangulanine, cocamidopropylbetaine, choline, and agmatine. Conversely, the accumulation of stepharine and shikonin in Chinese jujube and sour jujube was higher in the red stage than in the white stage. In the HW vs. SW comparison group, 21 DAMs were identified; compared with HW, 16 DAMs were up regulated and 5 were down regulated in SW. In the HR vs. SR comparison group, we identified 24 DAMs; compared with HR, 14 DAMs were up regulated and 10 DAMs were down regulated in SR. This also indicates that the accumulation of DAMs in sour jujube was higher than that in Chinese jujube; moreover, the accumulation was higher in the white ripening stage than in the red stage. These metabolites mainly include 3-hydroxypropylpalmitate glc-glucosamine, acetylcholine, Fer-agmatine, frangufoline, jubanineA, *N*-feruloylagmatine, *N*-p-coumaroyl agmatine, scutianineC, serotonin, and tryptamine. However, the accumulation of DAMs such as 6-deoxyfagomine, lotusine, and nuciferine was higher in Chinese jujube than in sour jujube.

**Figure 4:**
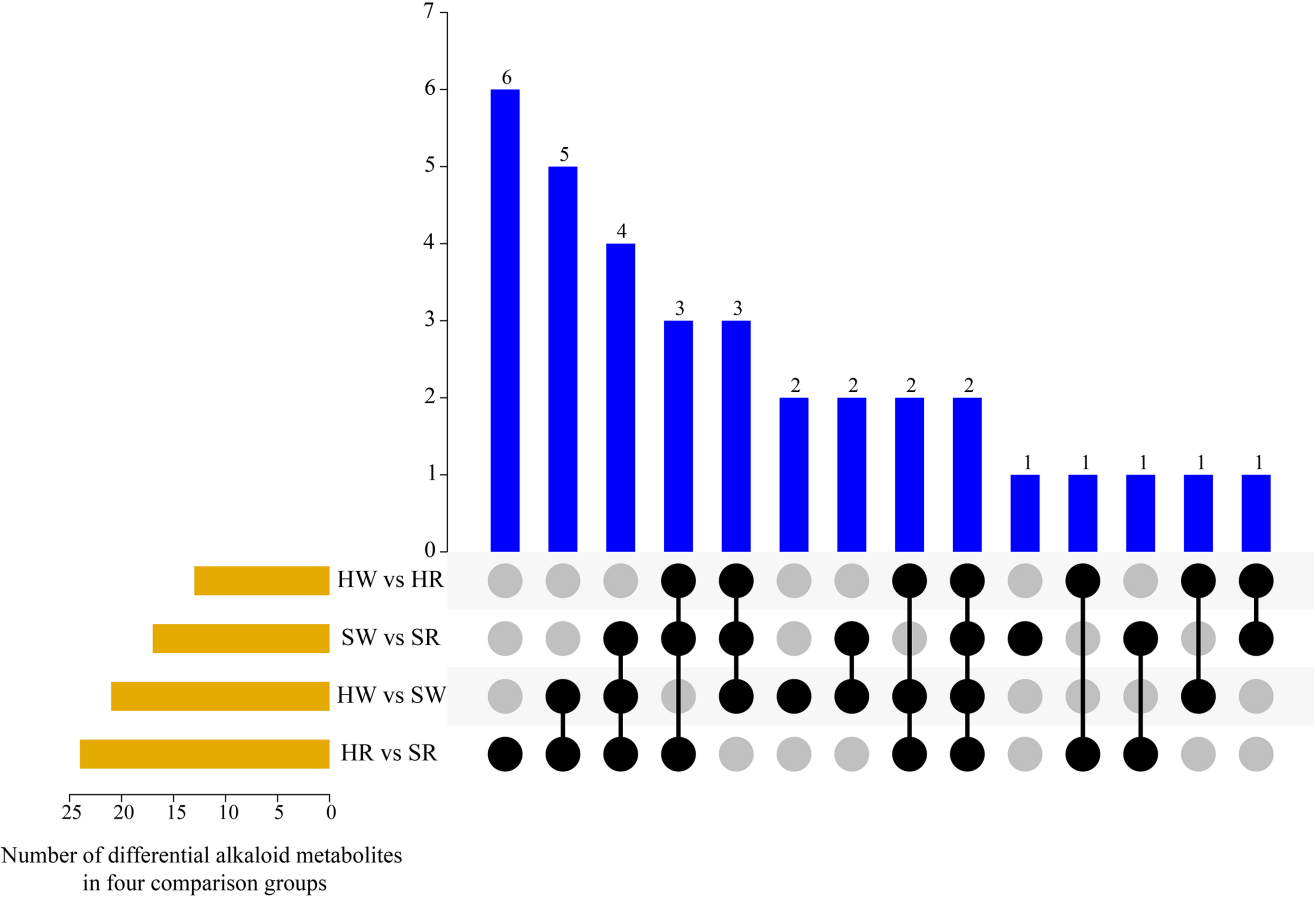
UpSet plot illustrating overlapping and specific alkaloid metabolites in various comparison groups. HW, HPZ white ripening stage; HR, HPZ red stage; SW, TGSZ white ripening stage; SR, TGSZ red stage.

By further analyzing the characteristics of DAMs in the different groups (**Figure 4**), we found that there were one to six metabolites that differed in one or more comparison groups; in particular, two metabolites, jubanine A and *N*-feruloylagmatine, differed in all comparison groups. In the SW vs. SR, HW vs. SW HR vs. SR comparison groups, there were one, two, and six DAMs with single differences, respectively (**Table 1**), but there were no single differential metabolites in the HW vs. HR comparison group.

**Table 1:** Alkaloid metabolites unique to each comparison group.

Comparison group	ID	Alkaloid metabolites	Class
SW vs SR	pmb1912	10-Formyl-THF	Alkaloids
HW vs SW	mws0191	Betaine	Alkaloids
	P2439	Norisoboldine	Alkaloids
HR vs SR	pme2268	Trigonelline	Alkaloids
	P3309	N-nornuciforine	Alkaloids
	Wmzzp003295	Yuzirine	Alkaloids
	pmp000459	Lirinidine	Alkaloids
	pmp000876	N-methylasimilobine	Alkaloids
	pmp000457	Nornuciferine	Aporphine alkaloids

### 3.3 Principal component analysis of DAMs of jujube fruits

**Figure 5:**
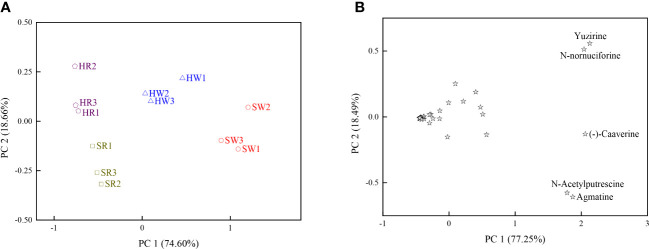
**(A)** Scatterplot based on the principal component analysis of DAMs. **(B)** Scatterplot based on the principal component analysis of the four samples. HW, HPZ white ripening stage; HR, HPZ red stage; SW, TGSZ white ripening stage; SR, TGSZ red stage.

Principal component analysis (PCA) was performed using 34 DAMs; two principal components, PC1 and PC2, explained 93.26% of the total variation (**Figure 5A**). The first principal component (PC 1) explained 74.60% of the total variation, and large positive values were associated with the relative content of *N*-acetylputrescine and agmatine, suggesting that these two metabolites contributed significantly to PC1.The second principal component (PC2) contributed 18.66% of the total variation, and large positive values were associated with the relative content of *N*-nornuciforine and yuzirine, suggesting that these two metabolites contributed significantly to PC 2. The white ripening stage and red stage of Chinese jujube and sour jujube were separated in the PCA scatterplot, mainly due to their differences in PC1 and PC2.

In scatter plots derived from the PCA of the DAMs based on the four samples, PC1 and PC2 together explained 95.74% of the total variation (**Figure 5B**). PC1 and PC2 explained 77.25% and 18.49% of the total variation respectively. Moreover, HW and SW positively contributed to PC1, HR positively contributed to PC2. The PC1 of *N*-nornuciforine, yuzirine, *N*-acetylputrescine, (-)-caaverine, and agmatine were higher and indicated higher contents of these five alkaloid metabolites in HW and SW. The PC2 of *N*-nornuciforine and yuzirine was higher and indicated higher content of these two metabolites in HR.

### 3.4 K-means cluster analysis of DAMs of jujube fruits

**Figure 6:**
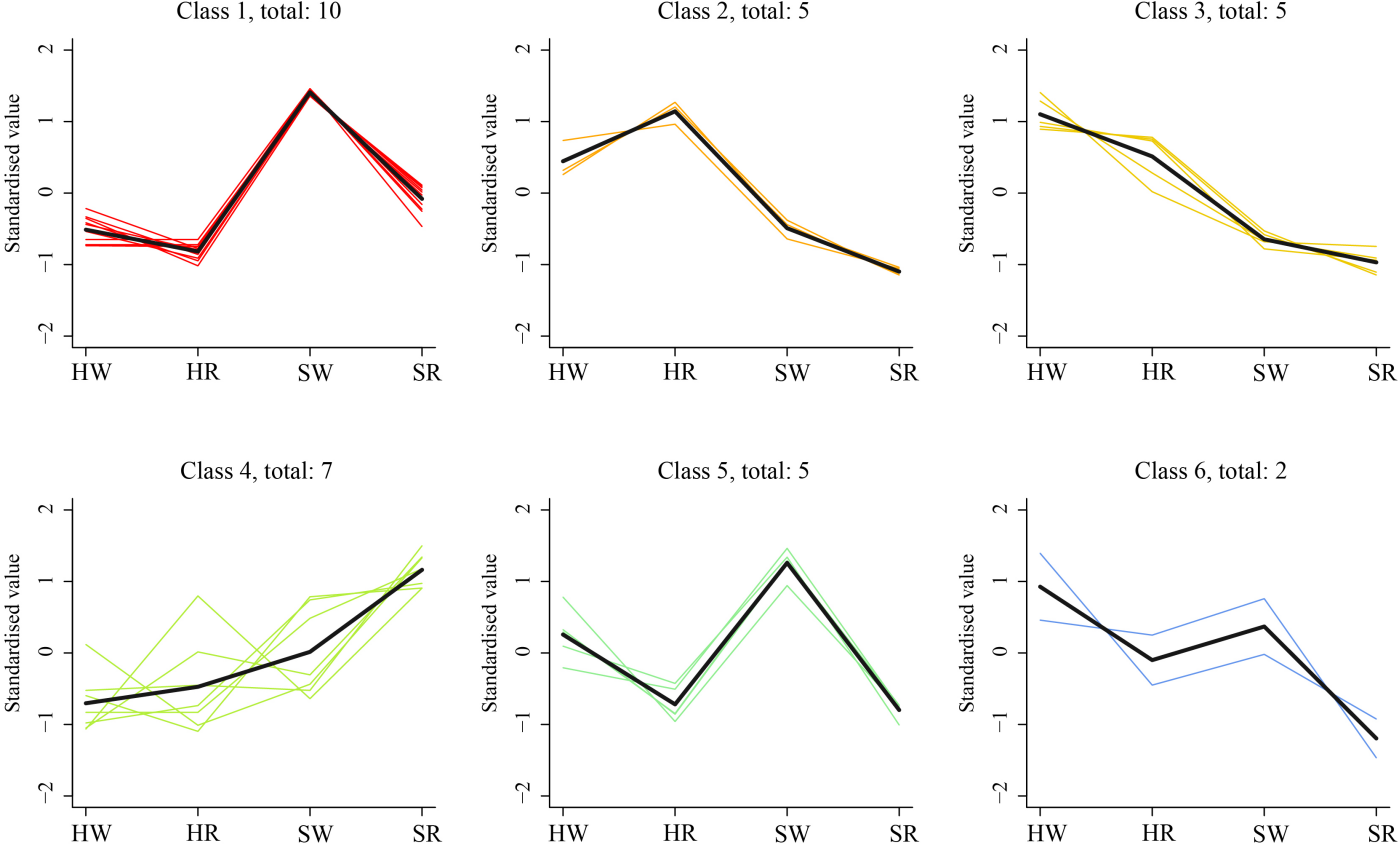
K-means cluster trend map of DAMs. HW, HPZ white ripening stage; HR, HPZ red stage; SW, TGSZ white ripening stage; SR, TGSZ red stage.

To further investigate the change characteristics of DAMs during different harvest stages in Chinese jujube and sour jujube, the relative content of alkaloid metabolites in all samples was standardized by z-value according to screening criteria, and analyzed by a k-average algorithm. The DAMs showed six change trends in the four samples (class 1–6; **Figure 6**). The number of metabolites classified in classes 1 and 5 were 10 and 5, respectively and showed the highest content in SW. Among them, jubanine A and *N*-feruloylagmatinein in class 1 showed evident differences among the four samples. *N*-acetylputrescineand agmatine in class 5 showed distinct differences in PCA. There were five DAMs in class 2, which showed the highest in HR, among them *N*-nornuciforine and yuzirine with obvious differences were classified class 2. Further, there were five metabolites in class 3, with descending order of concentration of HW > HR > SW > SR, indicating higher content of these metabolites in Chinese jujube than in sour jujube. Two metabolites, stepharine and shikonin, were classified as class 4, and were present at high levels in the red stage.

### 3.5 KEGG pathway analysis of alkaloid metabolism of jujube fruits during different harvest periods

**Figure 7:**
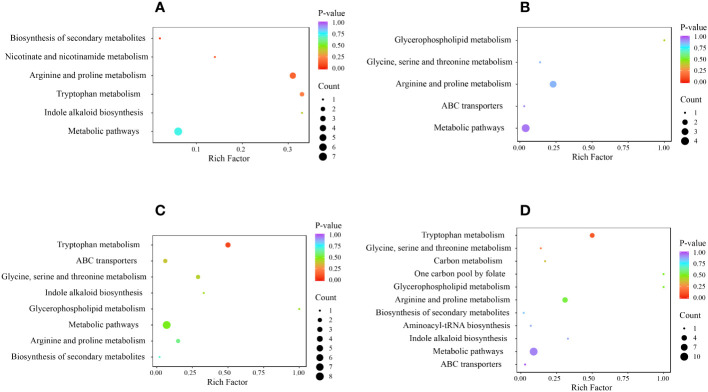
KEGG enrichment of DAMs in each comparison group, where **(A)** indicates HR vs. SR, **(B)** indicates HW vs. HR, **(C)** indicates HW vs. SW, and **(D)** indicates SW vs. SR. HW, HPZ white ripening stage; HR, HPZ red stage; SW, TGSZ white ripening stage; SR, TGSZ red stage.

Next, to investigate the metabolism of alkaloids, we performed KEGG pathway enrichment analysis and functional annotation of DAMs (**Figure 7**). The DAMs in the 4 comparison groups were enriched in 12 metabolic pathways, including glycine, serine, and threonine metabolism (ko00260); arginine and proline metabolism (ko00330); tryptophan metabolism (ko00380); glycerophospho lipid metabolism (ko00564); one carbon pool by folate (ko00670); nicotinate and nicotinamide metabolism (ko00760); indole alkaloid biosynthesis (ko00901); amino acyl-tRNA biosynthesis (ko00970); metabolic pathways (ko01100); biosynthesis of secondary metabolites (ko01110); carbon metabolism (ko01200); and ABC transporters (ko02010). All comparison groups had DAMs enriched in the ko00330 and ko01100 metabolic pathways, whereas those enriched in the ko00760 metabolic pathway only existed in the HR vs. SR comparison group. DAMs enriched in ko00670, ko00970, and ko01200 metabolic pathways were only present in the SW vs. SR comparison group.

### 3.6 Genes annotated to metabolic pathways based on transcriptome

**Figure 8:**
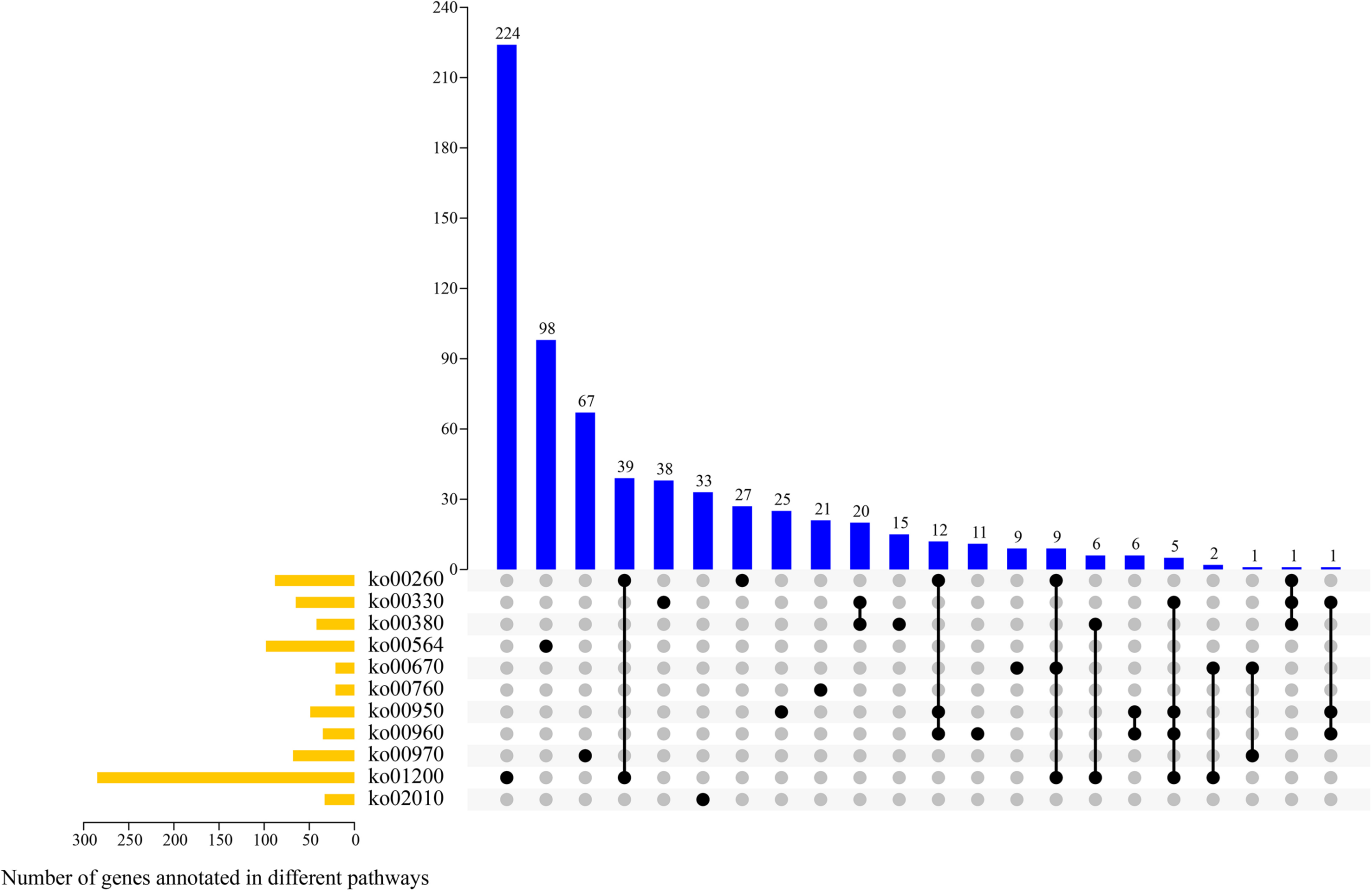
UpSet plot revealing overlapping and specific genes related to alkaloid metabolism pathways in four samples.

According to the transcriptomics data and KEGG enrichment characteristics of DAMs, 11 potential pathways involved in alkaloid synthesis and metabolism were screened out, and we analyzed the genes involved in their metabolic pathways (**Figure 8**). The 11 pathways were as follows: glycine, serine, and threonine metabolism (ko00260); arginine and proline metabolism (ko00330); tryptophan metabolism (ko00380); glycerophospho lipid metabolism (ko00564); one carbon pool by folate (ko00670); nicotinate and nicotinamide metabolism (ko00760), isoquinoline alkaloid biosynthesis (ko00950); tropane, piperidine, and pyridine alkaloid biosynthesis (ko00960); amino acyl-tRNA biosynthesis (ko00970); carbon metabolism (ko01200); and ABC transporters metabolism (ko02010). Overall, there were between one and 224 genes involved in one or more metabolic pathways. Most genes (n = 285) were enriched in ko01200 pathway, of which 224 genes were only involved in this pathway. Further, 98, 33, and 21 genes were enriched in ko00564, ko02010, and ko00760 pathways, respectively, and these genes were only involved in these enriched metabolic pathways.

### 3.7 Analysis of differentially expressed genes (DEGs) of jujube fruits during different harvest periods

**Figure 9:**
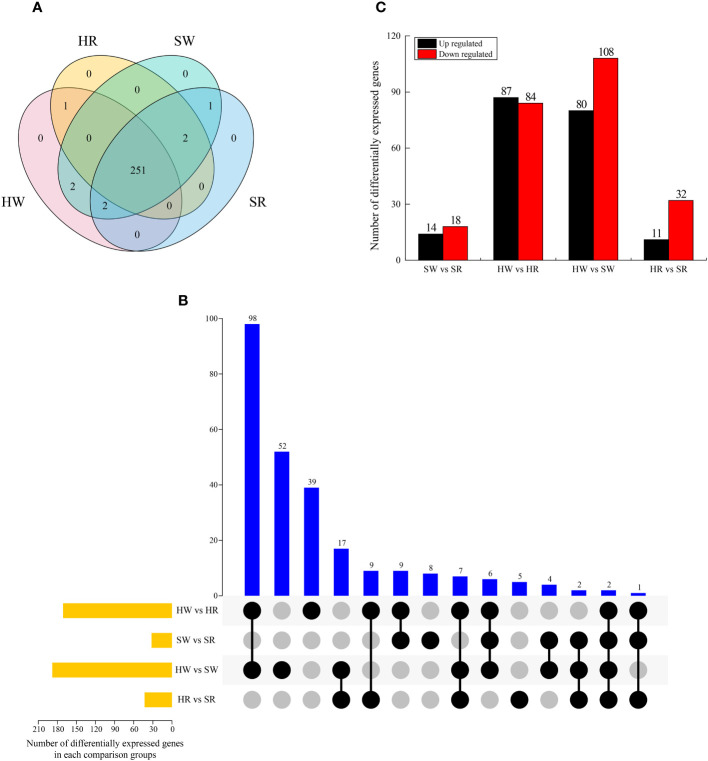
**(A)** Venn diagram of DEGs in four samples. **(B)** UpSet plot illustrating the overlapping and specific DEGs in each comparison group. **(C)** Upregulated and downregulated DEGs in each comparison group. HW, HPZ white ripening stage; HR, HPZ red stage; SW, TGSZ white ripening stage; SR, TGSZ red stage.

We analyzed the genes enriched in the metabolic pathway of alkaloids and screened the DEGs in four samples. A total of 259 DEGs were identified (**Figure 9A**), among which 251 were expressed in all samples; no DEGs were expressed in only one sample. *Gene 30628* was expressed in SW and SR samples, indicating that it was only expressed in sour jujube; conversely, *Ziziphus_jujuba_newGene_5605* was expressed in HW and HR, indicating that it was only expressed in Chinese jujube. Two genes, *gene32374* and *gene28293*, were expressed in HW and SW samples, indicating that they were only expressed in the white ripening stage.

By analyzing the DEGs in different comparison groups, we found that one to 98 genes were different in one or more comparison groups (**Figure 9B**). Two genes, *gene19198* and *gene15104*, were differentially expressed in all groups. Some DEGs only existed in a single comparison group. The highest number of DEGs was observed in HW vs. SW group (n = 52), whereas the lowest number of DEGs was noted in HR vs. SR group (n = 5). Further, the number of DEGs in the two comparison groups, SW vs. SR (32) and HR vs. SR (43), was relatively small (**Figure 9C**), whereas the other two comparison groups had a large number of DEGs, with 177 and 188 DEGs in HW vs. HR and HW vs. SW groups, respectively. Simultaneously, we analyzed the up regulation and down regulation of DEGs in each comparison group. The number of up regulated and down regulated genes showed a small difference in SW vs. SR and HW vs. HR groups, whereas it displayed a large difference in the HR vs. SR and HW vs. SW groups.

### 3.8 K-means cluster analysis of DEGs of jujube fruits

**Figure 10:**
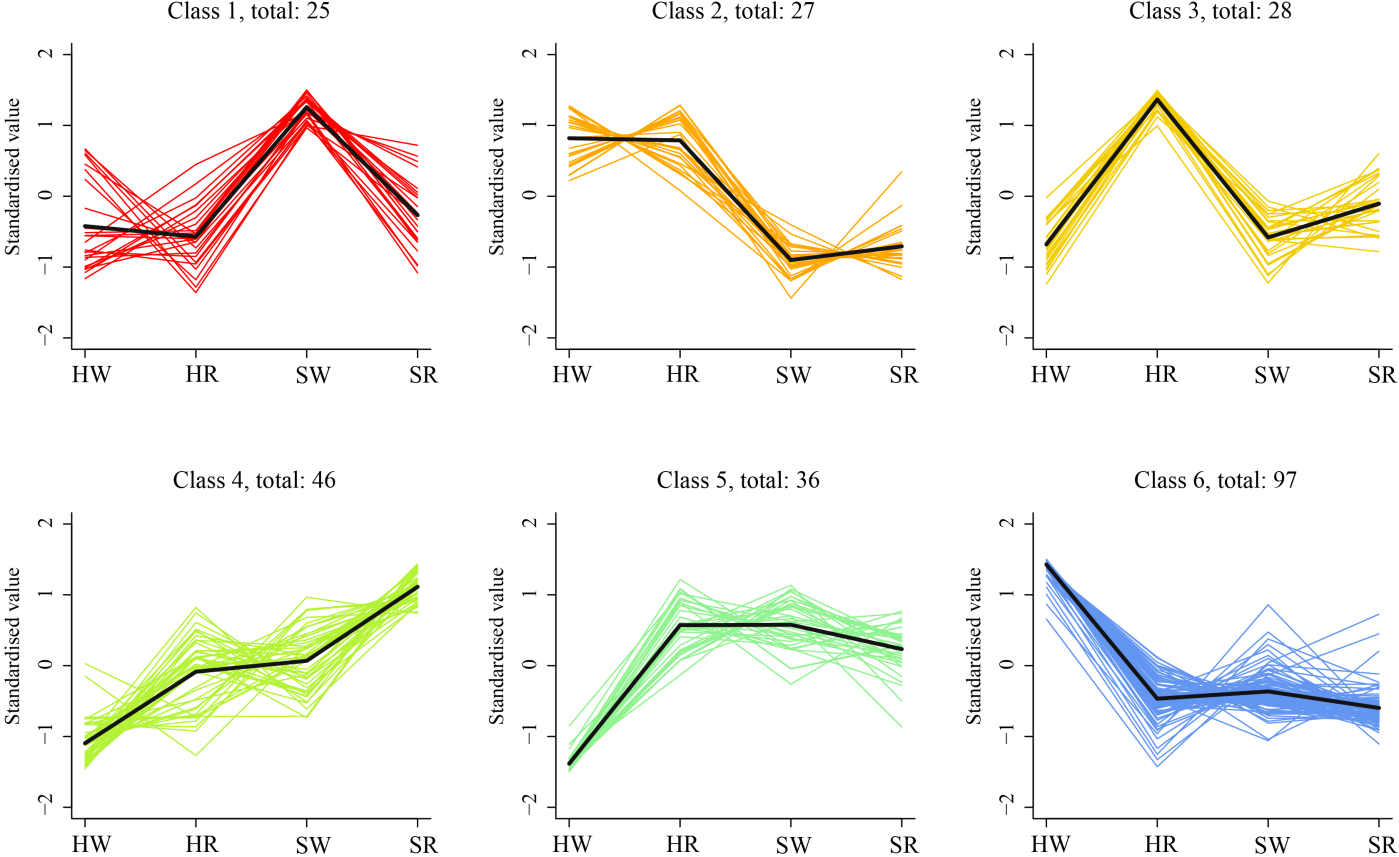
K-means cluster trend maps of DEGs. HW, HPZ white ripening stage; HR, HPZ red stage; SW, TGSZ white ripening stage; SR, TGSZ red stage.

To analyze the expression trends of DEGs in the different samples, we performed k-average algorithm analysis. The DEGs showed six change trends in the four samples class1–6 (**Figure 10**). The number of genes in each class was 25, 27, 28, 46, 36, and 97, respectively. The expression of DEGs in class1 was highest in SW, and in class3 was highest in HR. The expression levels of DEGs in HR, SW, and SR in classes 5 and 6 were similar. However, the expression of DEGs in class5 was lowest in HW, and in class6 was highest in HW. The expression levels of class2 genes were highest in HW and HR and lowest in SW and SR. The genes in class4 had the lowest expression in HW and the highest expression in SR.

### 3.9 Correlation analysis between DAMs and DEGs

**Figure 11:**
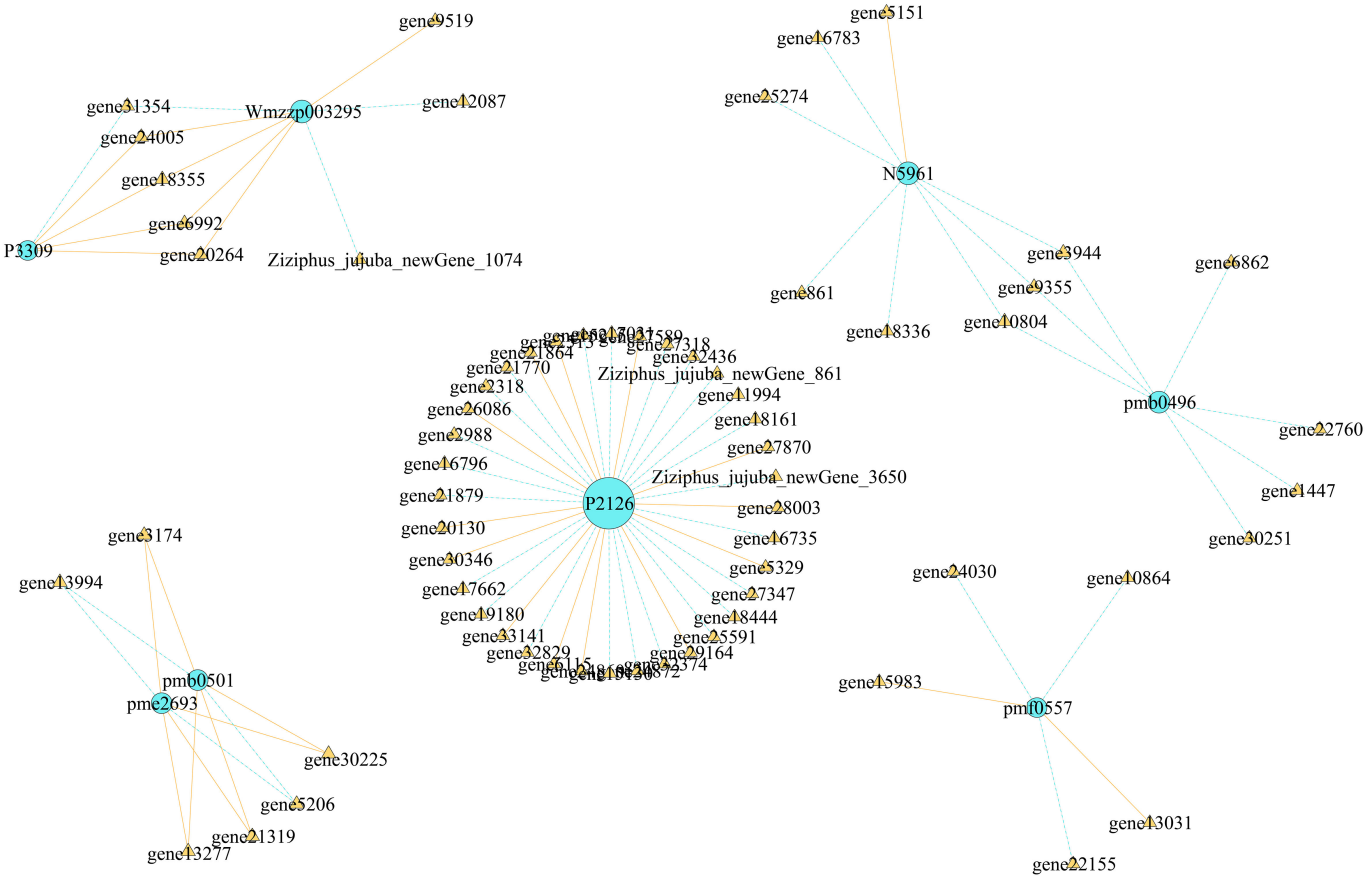
Regulatory network map of DAMs and DEGs.

We conducted correlation analysis of the important screened alkaloid metabolites and DEGs, revealing that each metabolite had multiple candidate genes that might be involved in regulating its metabolism (**Figure 11**). In total, 5 genes were associated with the change in shikonin (pmf0557) metabolism, and 36 genes were associated with the change in stepharine (P2126) metabolism, all of which were involved in the metabolism of a single substance. Six genes were found to be involved in the synthesis and metabolism of *N*-acetylputrescine (pme2693) and agmatine (pmb0501), three genes were involved in the synthesis and metabolism of jubanine A (N5961) and *N*-feruloylagmatine (pmb0496), and five genes were involved in the synthesis and metabolism of *N*-nornuciforine (P3309) and yuzirine (Wmzzp003295).

### 3.10 Analysis of candidate genes involved in alkaloid metabolism

**Figure 12:**
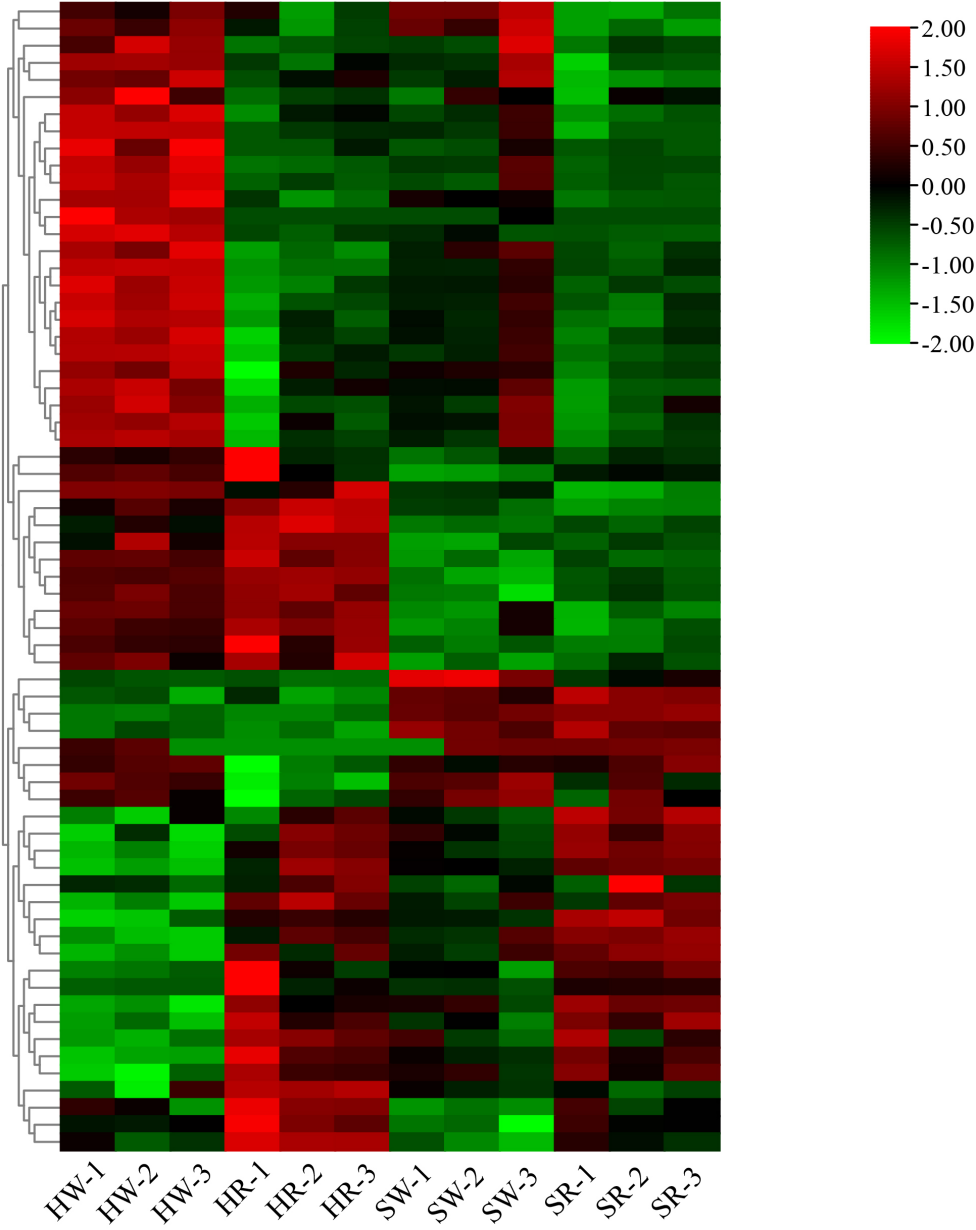
Heatmap of differentially expressed genes. HW, HPZ white ripening stage; HR, HPZ red stage; SW, TGSZ white ripening stage; SR, TGSZ red stage.

Finally, we constructed an expression heat map of DEGs related to important DAMs (**Supplement 3**; **Figure 12**). Based on the differences in their expression levels among different groups, 11 candidate genes, including *gene10804, gene12087, gene16783, gene18355, gene21879, gene22155, gene30225, gene30346, gene32374, gene5151*, and *Ziziphus_jujuba_newGene_1074*, were screened out. According to the gene annotation information, the 11 candidate genes were annotated as 1 *UGT74B1*, 1 *frmA*, 1 *pckA*, 1 *mmsA*, 1 *gcvH*, 1 *TR1*, 2 *cysK*, 1 *pfkA*, 1 *ALDH*, and 1 *accC* gene, respectively. Simultaneously, KEGG pathway annotation demonstrated that these genes were involved in the pathways related to alkaloid metabolism.

## 4 Discussion

Chinese jujube and sour jujube are the two most important species of the jujube genus, with alkaloid-rich fruits. *Ziziphus jujuba* ‘Hupingzao’ is a local variety in Taigu, Shanxi, China, and is a representative variety of Chinese jujube (Wang, 2015). There are many applications of sour jujube pulp, such as sour jujube powder, sour jujube cake, and sour jujube juice (Cui et al., 2016). Research on the synthesis and metabolism of alkaloids in sour jujube can facilitate its targeted application and improve the utilization efficiency of germplasm resources. Therefore, in this study, the representative variety *Ziziphus jujuba* ‘Hupingzao’ and the representative resource *Ziziphus spinosa* ‘Taigusuanzao’ were used as test materials to determine the mechanism of alkaloid synthesis and metabolism. The white ripening and red stages are the two main utilization stages of jujube fruits. Jujube fruits in the white ripening stage are generally used for preparing candied jujube (Chang, 2018), whereas those in the red stage are mainly used for the production of direct edible and dried jujube as well as beverages, jams, vinegar, wine, and other related processing products (Wang et al., 2022; Zahra et al., 2022). Hence, these two important harvest stages were selected in this study.

In this study, 44 alkaloid metabolites were identified from 4 samples based on widely targeted metabolomics and divided into 5 classes: alkaloids, aporphine alkaloids, isoquinoline alkaloids, phenolamines, and plumeranes. Previous studies have shown that different types of alkaloids have different bioactive functions. For example, isoquinolines constitute the largest group of alkaloid substances and have broad-spectrum antibacterial activity, especially against *Staphylococcus aureus* and methicillin-resistant *Staphylococcus aureus* (Chang and Dai, 2020). Indole alkaloids have antitumor effects (Bai et al., 2019). Three metabolites, namely, *N*-p-coumaroylagmatine, frangufoline, and scutianineC, were not detected in Chinese jujube fruit but only in sour jujube, and may be specific to the latter. For different species, most of the differential metabolites showed higher accumulation in sour jujube than in Chinese jujube, but only a few metabolites showed higher accumulation in Chinese jujube than in sour jujube, including 6-deoxyfagomine, lotusine, and nuciferine. In terms of harvest stage, most metabolites were higher accumulated at the white ripening stage than at the red stage, the opposite as true for a few substances such as stepharine and shikonin. 6-Deoxyfagomine has been reported to possess a strong antihyperglycemic activity (Im et al., 2013). Moroever, lotusine has been shown to exert antihypertensive effects (Zhan et al., 2023). Nuciferine and stepharine play important roles in the treatment of inflammation-related diseases (Hao et al., 2020; Zhao et al., 2023), and shikonin has been shown to exert anticancer effects (Qi et al., 2022). In addition, the main alkaloids components in different plants were different. For example, 1-deoxynojirimycin DNJ is the most marked piperidine alkaloid in mulberry (Cao, 2020); Benzyl isoquinoline alkaloids are the main active components in lotus leaf and lotus plumule (Wang L. et al., 2023). Therefore, for practical purposes, the stage or species with high accumulation of different substances should be selected to be used in a targeted way.

The DAMs were annotated to 12 metabolic pathways, and all 4 comparison groups had metabolites enriched in ko00330 and ko01100 pathways. These two pathways are speculated to be the most important or common pathways of alkaloid metabolism in Chinese and sour jujube. The metabolites enriched in the ko00760 pathway were only observed in the HR vs. SR group, indicating that this pathway is unique for alkaloid metabolism in Chinese jujube. The metabolites enriched in ko00670, ko00970, and ko01200 pathways were only observed in the SW vs. SR group, indicating that these pathways were unique for alkaloid synthesis in sour jujube. This may explain the higher alkaloid accumulation in sour jujube than that in Chinese jujube at both harvest stages.

Through combined transcriptomics and metabolomics analyses, in this study, we could not only identify common and unique DAMs and DEGs in each comparison group but also clarify the gene regulation of metabolites. We revealed candidate genes involved in the regulation of important metabolites; for instance, 5 genes were involved in the regulation of shikonin metabolism, and 36 genes were involved in the regulation of stepharine metabolism. This provides a theoretical basis for further research on the regulation of these genes encoding corresponding metabolites. Furthermore, we screened genes related to the regulation of multiple metabolite synthesis by a single gene. For example, five genes were involved in the synthesis and metabolism of *N*-nornuciforine (P3309) and yuzirine (Wmzzp003295). In summary, we screened out 11 important candidate genes involved in various pathways of alkaloid metabolism in jujube fruits. Among them, *UGT74B1* plays an important role in the process of glycosylation (Douglas Grubb et al., 2004; Lafite et al., 2019), and the function of other genes in jujube alkaloid metabolism is unknown. Therefore, in order to regulate the alkaloids metabolism in jujube, the precise role of these genes in the regulation of alkaloid metabolites needs to be further studied.

## 5 Conclusion

In this study, 44 alkaloid metabolites were identified in 4 samples, among which 3 metabolites, namely, *N*-p-coumaroylagmatine, frangufoline, and scutianine C, were unique to sour jujube fruit. We revealed that most DAMs showed higher accumulation in sour jujube than in Chinese jujube and that the accumulation was higher in the white ripening stage than in the red stage; however, the opposite was true for a few metabolites. Three metabolites (6-deoxyfagomine, lotusine, and nuciferine) showed higher accumulation in Chinese jujube than in sour jujube, and two metabolites (stepharine and shikonin) showed higher accumulation in the red stage than in white ripening stage. DAMs were annotated to 12 metabolic pathways, and transcriptomic data revealed that 259 DEGs were involved in alkaloid synthesis and metabolism. By mapping the regulatory networks of DAMs and DEGs, we screened out important metabolites and 11 candidate genes. This study preliminarily elucidated the molecular mechanism of jujube alkaloid synthesis.

## Data availability statement

The original contributions presented in the study are publicly available. This data can be found here: https://ncbi.nlm.nih.gov/bioproject/PRJNA1002126.

## Author contributions

XX: Formal Analysis, Writing – original draft. AZ: Funding acquisition, Writing – review & editing. YW: Data curation, Writing – original draft. HR: Data curation, Writing – original draft. WS: Investigation, Writing – original draft. YL: Methodology, Writing – original draft. MS: Data curation, Writing – original draft. LL: Methodology, Writing – original draft. DL: Funding acquisition, Writing – review & editing.
